# Robotic Surgery for Gastrointestinal Malignancies—A Review of How Far Have We Come in Pancreatic, Gastric, Liver, and Colorectal Cancer Surgery

**DOI:** 10.3390/cancers17233802

**Published:** 2025-11-27

**Authors:** Yael Weksler, Guy Lifshitz, Shmuel Avital, Yaron Rudnicki

**Affiliations:** Department of Surgery, Meir Medical Center, Gray Faculty of Medicine, Tel Aviv University, Kfar Saba 44281, Israel; guy.lifshitz@clalit.org.il (G.L.); shmuel.avital@clalit.org.il (S.A.); yaronru@clalit.org.il (Y.R.)

**Keywords:** robotic colorectal surgery, robotic gastric surgery, robotic pancreatic surgery, robotic liver surgery

## Abstract

Introduction: Robotic-assisted surgery offers technological advantages such as three-dimensional visualization and improved dexterity, yet its clinical adoption in gastrointestinal (GI) malignancies is supported by evidence of varying quality, consisting mainly of retrospective studies. This review provides a structured summary of the current evidence for robotic surgery in pancreatic, gastric, liver, and colorectal cancers. Methods: A comprehensive literature review was conducted to assess and summarize the perioperative, long-term, and oncological outcomes of robotic-assisted surgery compared to laparoscopic and open approaches for the aforementioned GI malignancies. Results: The application of the robotic platform is most advanced in colorectal surgery. High-quality evidence for rectal cancer demonstrates improved quality of mesorectal specimens, better preservation of urinary and sexual function, and lower local recurrence rates. Across all reviewed GI malignancies, robotic surgery consistently shows advantages in lower conversion-to-open rates, reduced intraoperative blood loss, and shorter hospital stays, though it is associated with longer operative times and higher costs. The evidence for pancreatic and liver surgery is less mature due to the complexity of these procedures. Data for gastric surgery suggests improved lymph node retrieval and, in one long-term study, better disease-free survival. Conclusions: The highest-quality evidence supports the robotic approach for rectal cancer, showing clear functional and oncological benefits. While several perioperative advantages are consistently reported across all GI sites, robust data demonstrating superior long-term survival are still limited for most procedures.

## Simple Summary

Robotic-assisted surgery provides advantages such as enhanced three-dimensional visualization and improved dexterity. However, its clinical adoption for gastrointestinal (GI) malignancies, including pancreatic, gastric, liver, and colorectal cancers, is mainly based on retrospective studies. This review summarizes the current evidence comparing robotic surgery to laparoscopic and open approaches in these cancers. While robotic surgery shows consistent benefits, such as lower conversion rates, reduced blood loss, and shorter hospital stays, it also involves longer operative times and higher costs. The strongest evidence is for rectal cancer, demonstrating improved mesorectal specimen quality, better preservation of urinary and sexual function, and lower recurrence rates. For other GI malignancies, long-term survival data remain limited.

## 1. Introduction

The need for the use of robots in surgery arises from two primary requirements: the capability for remote surgical intervention and the necessity to enhance accuracy and reproducibility. The first “robot surgeon” used on a human patient was developed by Victor Scheinman in 1978 and employed by Yik San Kwoh in 1985 to perform neurosurgical biopsies. The field of robotic-assisted surgery has evolved since then, and in 2000, the da Vinci Surgical System received approval from the U.S. Food and Drug Administration (FDA) for laparoscopic surgeries [1].

**Figure 1:**
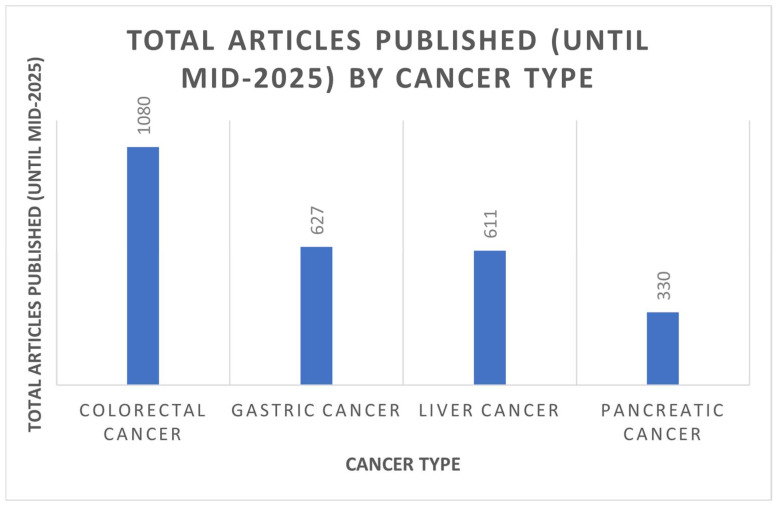
Total number of publications on robotic surgery for GI malignancies.

Robotic-assisted abdominal surgery benefits have already been widely recognized and characterized, which include three-dimensional visualization, improved dexterity with seven degrees-of-freedom, smooth motion, eliminating tremors, and scaling of motion, and may allow for tele-presence surgery [2]. It is thought that the advantages of robotic surgery, although substantial in volume, are mainly retrospective, observational, or based on surgeons’ experience, with only a limited number of randomized controlled trials (RCTs) [3,4,5,6,7,8,9,10,11,12,13,14,15,16,17,18,19,20,21] (Table 1). This review aims to provide a structured summary of the evidence currently available on robotic-assisted surgery for gastrointestinal malignancies, such as pancreatic, gastric, liver, and colorectal cancer (Figure 1) [22].

## 2. Robotic Surgery for Pancreatic Cancer

The data on robotic surgery for pancreatic cancer is relatively limited. From 2009 to 2025, there were just over 330 articles published regarding pancreatic cancer and robotic surgeries, with most of them (67%) published after 2019 [22]. The paucity of data may be attributed to pancreatic surgery being one of the most complicated subfields of general surgery. This complexity is due to the retroperitoneal location of the pancreas and its proximity to major vasculature [23]. Additional concerns have been raised concerning the approach’s cost-effectiveness and safety, especially during scenarios that require urgent conversion to open [24]. The most common robotic surgical procedures adopted were Robotic Distal Pancreatectomy (RDP) and the Robotic Pancreaticoduodenectomy (RPD) [25]. The first published RPD was performed in 2001 by Giulianotti [26], followed by the first RDP in 2003 [27].

For pancreatoduodenectomy, according to the National Cancer Database in the United States, the percentage of patients receiving a robotic approach increased from 1.1% to 7.5% between 2010 and 2019. In the same period, the percentage of robotic distal pancreatectomies rose from 2.2% to 19.4% [24].

### 2.1. Advantages of Robotic Surgery for Pancreatic Cancer

The morbidity around pancreatic surgery is as high as 68.7%, mainly delayed gastric emptying, pancreatic fistula, and post-pancreatectomy hemorrhage [28]. Some of the morbidity and challenges post-operatively may be attributed to the open approach or limitations of laparoscopy. The advantages of the robotic approach for pancreatic cancer revolve around the combination of a minimally invasive approach like laparoscopy and the added freedom of motion and maneuverability, like in open surgery. The articulated robotic hook provides a great advantage over other approaches in clearing all perineural tissue and lymphatics surrounding the aorta and celiac trunk. It provides surgeons with a way to carefully dissect the tail and the various vessels involved in a minimally invasive approach. The other endo-wristed instruments allow great maneuverability around the neck and tail of the pancreas, with added depth of visibility with the 3D vision [23].

The main robotic advantages that were found in the literature were a lower rate of conversion to open surgery (5–12.9% in comparison to 12–15.8%), improved spleen and splenic vein preservation (73–91.9% in comparison to 39–68%), and reduced hospital stay (a decrease of 1–2 days). As well as significantly lower estimated blood loss (150–400 vs. 300–775 mL), and decreased incisional SSI rate (7.9% vs. 10.1%) [23,29,30,31,32,33]. Da Dong et al. compared robotic vs. laparoscopic pancreaticoduodenectomy, and Daouadi et al. compared robotic vs. laparoscopic distal pancreatectomy; both have shown that the robotic arm had superior oncologic outcomes, with a higher rate of negative margins (84.4–95% vs. 80.1–83%) and improved lymph node yield (13–20 vs. 9–15) [32,34] (Table 2). Nassour et al. reported a significant advantage in overall survival for robotic distal pancreatectomy, with a median survival of 35.3 months, compared to open distal pancreatectomy, with a median survival of 24.9 months, based on data from the American National Cancer Database comprising 2718 patients. There was no significant difference in survival for pancreaticoduodenectomies.

Regarding robotic total pancreatectomy surgery, there is very limited data available in the literature; however, this type of surgery is also reported to be associated with shorter hospital stays. Additionally, it is described as having lower mortality rates (30 days: 2 vs. 4.8%, respectively, and 90 days: 4.3 vs. 9.4%) [35]. These reports need to be taken with a grain of salt, as they are mainly non-randomized, retrospective studies.

### 2.2. Disadvantages of Robotic Surgery for Pancreatic Cancer

Open or Laparoscopic Pancreatic surgery are by themselves prolonged procedures. The added operative time of the robotic approach is a major disadvantage from a clinical point of view as well as a financial one. The overall higher cost of the robotic surgery is another major deterrent for implementing this approach [3]. Although there are many advantages shown in the robotic pancreatic surgery, no significant difference in postoperative pancreatic fistula was reported. This can be explained by understanding that the current robotic platforms do not add any innovative new technology in the pancreas resection and stapling that may reduce pancreatic leak. There were also no advantages seen in rates of delayed gastric emptying and in reoperation rates, leaving much room for improvement [32].

### 2.3. Quality of the Data

Short and long-term outcomes of robotic pancreatic surgery compared to laparoscopic and open procedures are based mainly on meta-analyses and retrospective studies, with very few RCTs. To date, only three main RCTs have been published, comparing the robotic to the laparoscopic or open approach. The DIPLOMA [3] and LEOPARD [4] studies investigated Distal Pancreatectomy, while the EUROPA [5] study focused on Pancreatoduodenectomy.

The DIPLOMA trial was an international randomized study comprising 35 centers in 12 countries, and 131/127 patients in each arm (minimally invasive vs. open), originating from Italy, with a primary endpoint of radical resection (R0), defined as the minimum margin of ≥1 mm between tumor and surgical margin in Distal Pancreatectomy. The trial demonstrated noninferiority in radical resection rates and lymph node yield, along with comparable postoperative time to recovery and overall survival. Length of ICU admission was three days shorter after minimal invasive distal pancreatectomy in comparison to open surgery. This was the only postoperative factor significantly different between the groups. Nevertheless, there is no distinction made between laparoscopic and robotic surgeries in relation to the aforementioned outcomes.

Similarly, the LEOPARD trial was a multicenter randomized controlled study conducted in the Netherlands comparing minimally invasive distal pancreatectomy (MIS) with the open approach. The MIS group showed reduced intraoperative blood loss, lower rates of delayed gastric emptying, and shorter time to functional recovery, while achieving an equivalent rate of radical resection. However, it should be noted that only five patients in this trial underwent robotic surgery, with the remainder treated laparoscopically. The subsequent LEOPARD-2 trial compared open and laparoscopic pancreaticoduodenectomy (49 and 50 patients, respectively). Although the difference did not reach statistical significance, laparoscopic pancreaticoduodenectomy was associated with a higher number of complication-related deaths, and no difference was observed between the groups regarding time to functional recovery.

Due to those findings, the trial was prematurely terminated. According to many MIS pancreatic surgeons, the LEOPARD 2 [36] trial represents the swan song of laparoscopic pancreatoduodenectomy surgery and brings robotic pancreatoduodenectomy to the forefront [37].

The EUROPA trial enrolled 81 patients to compare robotic and open pancreaticoduodenectomy, with postoperative morbidity as the primary endpoint. The investigators reported higher hospital costs and increased rates of clinically relevant pancreatic fistula and delayed gastric emptying in the robotic group compared with the open group. Two randomized controlled trials, DIPLOMA-2 and PORTAL, are currently underway to expand the evidence base comparing MIS and open pancreaticoduodenectomy. Preliminary results from DIPLOMA-2 have shown non-inferiority of the MIS regarding postoperative complication index, alongside shorter hospital stay, less blood loss, and lower incidence of Grade B/C postoperative pancreatic fistula compared with open surgery [24].

From a technical standpoint, the extent of robotic assistance in pancreaticoduodenectomy varies among surgeons: some employ a hybrid robotic–laparoscopic technique, while others perform the entire procedure robotically [23]. For surgeons already proficient in laparoscopic pancreatectomy, approximately 10–20 consecutive cases are suggested to overcome the learning curve for robotic distal pancreatectomy, whereas achieving competency in robotic pancreaticoduodenectomy may require 25–50 cases [24,33]. Robotic surgery for pancreatic cancer is still in its infancy, and many more publications and technological advancements are needed before the true implementation of this approach in the common armamentarium of Hepatobiliary surgeons.

## 3. Robotic Surgery for Gastric Cancer

Gastrectomy with D2 lymphadenectomy is the standard surgical treatment with curative intent for patients with gastric cancer. The first reported Robotic Gastrectomy was performed by Hashizume et al. in 2003 [38], and since then its use has been steadily increasing. From 2009 to mid-2025, 627 articles were published concerning robotic surgeries in gastric cancer [22]. Since 2015, there has been a 74% rise in the number of publications, with the most recent papers being reported mainly from Japan and China [39].

### 3.1. Advantages of Robotic Surgery for Gastric Cancer

Reported advantages of the robotic approach for gastric cancer include lower intraoperative blood loss (24–229 mL vs. 46–288 mL), shorter length of hospital stay (mean difference of 0.5 day), shorter time to first flatus (mean difference of 0.2 day), and shorter time to first oral intake (mean difference of 0.2 day) [8,40,41]. An advantage in surgical morbidity and oncologic outcomes is still questionable. There was a reported trend towards retrieving a greater number of lymph nodes in some studies (34.5–36 vs. 26.6–30 nodes) [40,41,42], while others found similar yields between the two groups [5,43]. Nevertheless, Lu et al. demonstrated a higher number of extraperigastric lymph nodes retrieved in the robotic distal gastrectomy group (17.6 vs. 15.8). Moreover, patients in the robotic group were more likely to initiate adjuvant chemotherapy earlier (on postoperative day 28 vs. 32) [6].

The advantages of the robotic approach are attributed to its three-dimensional magnified view and enhanced instrument articulation, which may facilitate more delicate tumor dissection along optimal anatomical planes while minimizing excessive tissue traction. In addition, the robot’s tremor-filtering system, unlike conventional laparoscopy, can help prevent inadvertent vascular injury and reduce overall surgical trauma. These advanced capabilities may lower the risk of intraoperative dissemination of circulating tumor cells and attenuate systemic inflammatory responses, ultimately contributing to better recovery, improved prognosis, and reduced risk of tumor recurrence [38,41]. The learning curve for robotic gastric surgery proficiency seems shorter. It has been found that fewer cases are required to achieve proficiency compared to laparoscopic surgery, and the number ranges from 6 to 25 surgeries per surgeon, in comparison to 40 to 100 cases for the laparoscopic approach. This outcome is particularly pronounced among surgeons with laparoscopic experience [41,44].

Data on the impact of robotic surgery stratified by the extent of gastric surgery is limited. The difference in complexity of partial gastrectomy and total gastrectomy might warrant further investigation. Total gastrectomy with an esophago-jejunal anastomosis is considered a much more challenging procedure with a higher risk for anastomotic leakage due to the limitation of performing an anastomosis at the level of the diaphragmatic hiatus and the mediastinum. Only one study was found to have a subgroup analysis by type of gastric resection performed [45]. In this analysis, comparison of the two types of gastrectomies performed via the robotic versus the laparoscopic approach demonstrated a longer operative time overall. Specifically, in distal gastrectomy, the robotic group exhibited lower intraoperative blood loss and a greater number of lymph nodes retrieved, whereas in total gastrectomy, the robotic approach was associated with a shorter time to first flatus. It is the author’s personal experience that using the robotic platform for esophago-jejunal anastomosis is much easier and safer, but the data to support this claim is missing.

In 2022, two prospective pioneering studies comparing robotic and laparoscopic total radical gastrectomy for advanced gastric cancer were published by Chen et al. [46] and Li et al. [47]. Together, these studies included 286 patients and demonstrated that robotic total gastrectomy offers advantages such as reduced intraoperative blood loss (38–110 vs. 66–150 mL), faster postoperative recovery with less surgical trauma, and a higher number of retrieved lymph nodes (43–51 vs. 35–45).

### 3.2. Disadvantages of Robotic Surgery for Gastric Cancer

Similarly to other abdominal robotic surgeries, the main disadvantages found in gastric robotic surgery include longer procedural time (33 to 44 min longer) and higher costs (3913 USD higher), with the comparative relative mortality being similar to laparoscopy [38,41].

### 3.3. Quality of the Data

To date, only four randomized controlled trials have reported comparisons between robotic gastrectomy and laparoscopic gastrectomy. One trial, published in Chinese [7], examined patient-reported outcomes and demonstrated superiority of the robotic approach in domains of general health, emotional well-being, and social functioning.

Two articles were published by Lu et al. based on the same RCT that included 300 patients in China who underwent distal gastrectomy, with reports published in 2021 (short-term outcomes [6]) and 2024 (long-term outcomes [8]). The short-term analysis found that the robotic approach was associated with faster postoperative recovery, lower morbidity, retrieval of a greater number of lymph nodes, and earlier initiation of adjuvant therapy (28 vs. 32 days). In line with these findings, the long-term analysis, with a three-year follow-up, demonstrated significantly higher disease-free survival (DFS) in the robotic group (85.8% vs. 73.2% for 3 years).

The fourth RCT, performed in Japan and published by Ojima et al. in 2021, included 241 patients undergoing total, proximal, or distal gastrectomy [9]. This study reported a lower incidence of postoperative complications of Clavien–Dindo grade II or higher (8.8% vs. 19.7%). However, it should be noted that in the analysis of each specific type of complication, no statistically significant difference was found between the groups. The researchers also reported a shorter time to first flatus and lower postoperative analgesic requirement in the robotic surgery group.

## 4. Robotic Surgery for Liver Cancer

The first robotic-assisted liver resection was reported by Ryska in 2006 [48]. Ever since, the field has evolved, and the robotic approach for liver resection is now employed in the treatment of malignant diseases with indications such as hepatocellular carcinoma (HCC), intrahepatic cholangiocarcinoma, and hepatic metastases from other origins [33].

Laparoscopic advancement in the field of liver surgery has been slower compared to other surgical fields, primarily due to technical challenges in hemorrhage control and difficulties in accessing the posterosuperior liver segments with laparoscopic instruments. Consequently, the progression of robotic liver surgery has also been relatively slow [49]. However, the advantages of the robotic platform—such as enhanced visualization and increased degrees-of-freedom—have enabled surgeons to address these challenges more effectively.

From 2006 to mid-2025, 611 articles were published concerning robotic liver resection (for all indications, not just cancerous). The most significant increase in the number of publications occurred between 2018 and 2019, with a 3-fold increase [22].

### 4.1. Advantages of Robotic Surgery for Liver Cancer

The vast majority of the studies published to date on robotic liver resection are retrospective. The four main meta-analyses were conducted in China, the Netherlands, and Italy, each comprising 12–31 studies [49,50,51,52]. The advantages of the robotic approach that have been conclusively demonstrated are: lower rate of conversion to open (2.1–4.7% vs. 8.5–12%), lower intraoperative blood loss (average difference of 53 mL), shorter hospital stay (an average of a one-day difference), lower overall morbidity (17.8% vs. 26.7%) and severe morbidity rate (3.9% vs. 7.9%), and higher rates of R0 resection. Di Sandro et al. [50] demonstrated similar overall survival between the groups, but with significantly better recurrence-free survival for the robotic group (46.8% vs. 24% for 5 years).

### 4.2. Disadvantages of Robotic Surgery for Liver Cancer

According to Pilz da Cunha et al. [49], the robotic group experienced a higher rate of hospital readmissions, a difference that reached significance only in the subgroup undergoing minor anterolateral resections. This may be attributed to a shorter length of hospital stay observed in this group, although statistical significance was not demonstrated. Several, though not all, studies demonstrated a prolonged operative duration in the robotic approach groups (240 vs. 190 min) [50,51]. There were no significant differences in blood loss, transfusion, Pringle use, surgical costs, and mortality.

### 4.3. Quality of the Data

In 2023, Di Benedetto F et al. [53]. reported the largest western experience of robotic liver resection for HCC, including 398 patients. In their retrospective cohort study, compared with open liver resection, robotic liver resection performed in tertiary centers represents a safe treatment strategy for patients with HCC and those with compromised liver function while achieving oncologic efficacy.

To date only one RCT comparing robotic liver resection to laparoscopic liver resection has been published, the ROC’N’ROLL [10] trial, which was conducted in Germany between 2022 and 2023, and included 80 patients. There was no significant difference between the groups in perioperative outcomes and quality of life within 90 days after the operation, as measured by patients’ questionnaires.

## 5. Robotic Surgery for Colorectal Cancer

Robotic Colorectal surgery is considered the most highly developed area for the application of robotics in general surgery. In the scientific literature, it is often difficult to separate studies addressing colonic tumors from those focusing on rectal tumors, as many investigations include both types collectively. Having said that, it is a well-known fact that rectal surgery is a much more challenging fit than surgery for colonic tumors and requires advanced surgical skills. In total, by mid-2025, 1080 articles addressing the robotic approach in both fields have been published [22].

The rapid evolution of this field is likely attributable to the predominant use of robotic systems in the pelvis, as is the case in Urological and Gynecological pelvic surgery. The pelvis is characterized by limited anatomical space, where the superior flexibility and range of motion offered by robotics are especially advantageous compared to laparoscopy. The rectum’s anatomical location within the pelvis, close to urogenital organs and surrounded by critical muscles and nerves responsible for continence and sexual function, presents significant challenges for conventional laparoscopy. Robotic systems, with their enhanced precision, are proposed to reduce intraoperative complications and conversion rates, while improving both short- and long-term clinical and oncological outcomes. However, most studies to date are retrospective and non-randomized, with inconsistent outcome definitions, a lack of long-term oncological data, and variable segmental stratification. Furthermore, the predominance of retrospective cohort studies in meta-analyses limits the overall strength and reliability of the evidence [54].

### 5.1. Advantages of Robotic Surgery for Colorectal Cancer

Published data have demonstrated that the proven benefits of robotic surgery for colon resection include: reduced conversion rates to open (3.6–6.1% vs. 9.4–11.1%), shorter hospital stay with an average difference of less than one day, reduced intraoperative blood loss (53.6 mL vs. 63.6 mL), earlier return of bowel movements (an average difference of 0.5 day), and even in some studies a higher lymph node yield (27 vs. 24) [55,56,57,58,59,60].

A potential advantage of the robotic approach is the ease in using robotic systems in performing intracorporeal bowel anastomoses, allowing the specimen to be retrieved through a smaller Pfannenstiel incision. This approach has the potential to reduce postoperative ileus, alleviate pain, and lower the risk of incisional hernias as a long-term complication. [61]. The data for rectal robotic surgeries suggest far fewer conversion rates (0–10% vs. 4–23%), shorter length of stay (1–3 days shorter), and shorter time to first flatus (mean difference of 1 day). There is no significant difference in overall postoperative complications [62,63,64,65,66].

Currently, total mesorectal excision (TME) combined with neoadjuvant chemoradiation is the standard treatment for locally advanced rectal cancer. However, TME for lower rectal cancer remains challenging even for experienced surgeons, particularly in patients with a narrow pelvis, male sex, obesity, anteriorly located lesions, bulky tumors, or those who have undergone neoadjuvant chemoradiotherapy. It is well established that the greatest benefit of the robotic approach in rectal cancer is seen in tall male patients with a narrow pelvis, obese individuals, and those with low rectal tumors [54]. Furthermore, despite complete mesorectal resection, preserving the superior hypogastric, inferior hypogastric, and pelvic nerves-critical for urinary and sexual function-remains technically demanding. For these reasons, robotic techniques have gained considerable recognition in performing TME for lower rectal cancers [67]. However, evidence regarding long-term oncological outcomes remains limited.

### 5.2. Disadvantages of Robotic Surgery for Colorectal Cancer

The robotic approach was associated with longer operative times (206 vs. 168 min) [55,56,57,58,60] and higher costs (surgery-specific costs of 8156 Euros vs. 3900 Euros, total costs 10,306 Euros vs. 7647 Euros) [57].

To date, only two RCTs have been published, focusing mainly on robotic colon resection: Jimenez et al. published in Spain in 2011 a study that included a total of 56 patients with sigmoid or rectal cancer and compared robotic and laparoscopic resections. There was no significant difference in complication rate, but the robotic operative time was longer. The distal resection margin and the number of lymph nodes obtained were greater in the specimen obtained using robotic surgery [12]. Another RCT, hailing from South Korea, investigated 71 patients with right colonic cancer. The study did not show the robotic approach to be superior to conventional laparoscopy regarding complication rates, postoperative pain, hospital stay duration, or tumor-free resection margins. However, operative time was significantly longer and associated costs were higher in the robotic group [11].

### 5.3. Quality of the Data

There is a substantially greater number of RCTs investigating robotic rectal surgery, likely attributable to the claimed advantages of robotic techniques in pelvic surgery as previously described. The two largest are the ROLARR and REAL trials: The ROLARR trial, from 2017, compared a robotic approach with laparoscopic surgery in patients undergoing rectal cancer resections. It included 471 patients from 10 countries and showed no significant difference in conversion to open rates, intraoperative complications, postoperative complications, quality of anatomical plane of surgery, 30-day mortality, bladder dysfunction, and sexual dysfunction. Subgroup analyses hinted at possible lower conversion rates for patients with obesity or male patients, but the trial was not powered to detect significant differences in these patient groups [13].

The REAL study from 2022 included 1240 patients with middle or lower rectal cancer in China. In relation to the short-term outcomes. The robotic group had less positive circumferential resection margins, fewer postoperative complications, better postoperative gastrointestinal recovery, shorter postoperative hospital stay, fewer abdominoperineal resections, fewer conversions to open surgery, less estimated blood loss, and fewer intraoperative complications than patients in the laparoscopic group. The long-term outcomes data from the REAL study were published in 2025: a 3-year cumulative incidence analysis showed that the locoregional recurrence rate was significantly lower in patients undergoing robot-assisted surgery compared to laparoscopic surgery (1.6% vs. 4.0%). These patients also had significantly improved disease-free survival rates (87.3% vs. 83.6%), although there was no significant difference in overall survival. The improved oncological outcomes following robot-assisted surgery were accompanied by significant enhancements in urinary function, as well as male and female sexual function scores, with no significant differences in chronic pain reported at any postoperative time point [14,15]. Willis et al. referred to the results of the REAL trial in their review, noting that this study had a larger sample size compared to earlier single-center studies. They emphasized that the trial exclusively included patients with tumors located in the mid and lower rectum, where the technical advantages of robotic systems are believed to be most pronounced [54]. Furthermore, the results of this study have yet to be incorporated into published reviews, and no meta-analyses have performed subgroup analyses specifically focused on this clinical context.

At least six additional RCTs have compared robotic and laparoscopic approaches for rectal surgery, comprising 668 patients [16,17,18,19,20,21]. The robotic approach has shown several advantages, including a lower conversion rate to open surgery (0–4.8% vs. 1.4–11%) and decreased hospital length of stay (0.5–2 days difference). There was no significant difference in blood loss intraoperatively. Interestingly, some of the studies exhibited longer operating time for the robotic approach, while others demonstrated a shorter operating time, specifically regarding TME [16]. According to Kim et al., sexual function 12 months postoperatively was better in the robot-assisted group than in the laparoscopic group [19]. Regarding oncological benefits, DeBakey et al. demonstrated significant improvement in distal margin acquisition (2.8 vs. 1.8 cm) [19], and Kim et al. exhibited a better lymph node harvest (18 vs. 15) [19]. Patriti et al. suggested a superior disease-free survival (DFS) with the robotic approach. However, certain trials did not identify any statistically significant differences between the two groups in key outcomes [16]. It is the personal experience of the authors that in advanced surgical centers, where robotic surgery is well established, it has become the gold standard of rectal surgery, with very few cases performed laparoscopically, but data supporting this claim are missing.

## 6. Discussion

Robotic surgery for GI malignancies has taken its place in the armamentarium of the general surgeon, as shown by the plethora of publications. Yet, the quality of the data is arguably very low, and as such, the strength of the proposed advantages is fairly weak: RCTs are lacking, as is sufficient data on long-term outcomes. There is significant heterogeneity among existing studies and differences in surgeon experience. Additionally, publication bias may obscure unfavorable results. This review has shown that the assimilation of the robotic approach into practice has different rates of advancement, with robotic colorectal surgery leading the charge, followed by robotic gastric surgery, robotic pancreatic surgery, and finally robotic liver surgery. There is a dichotomy between the increasingly very high volume of abdominal robotic surgery performed in the world and the very low volume of evidence of its advantage or superiority over laparoscopic or open surgery. The most common explanation for this contrast is usually attributed to external industry influences and marketing forces. Another possible explanation for this gap may be the less spoken, less reported, vast personal advantages for the robotic surgeons themselves. It has been demonstrated that the intense physical demands of surgical work—involving prolonged standing during operations, repetitive physical motions, and suboptimal ergonomic conditions—expose surgeons to work-related musculoskeletal disorders. These physical stressors not only induce pain and discomfort but also contribute to cumulative fatigue, diminished job satisfaction, and a gradual decline in mental resilience, ultimately resulting in professional burnout [68]. Emerging evidence indicates that robotic-assisted surgery contributes to a reduction in professional burnout among surgeons [69,70].

It is the author’s personal opinion that surgeons who transition into robotic surgery seldom revert back to laparoscopy if they have the chance. The added ease of the robotic platform, with 3D vision, a stable camera operated by the surgeon, multiple instruments with anti-tremor capabilities, and being able to sit while performing abdominal surgery and avoid standing for long hours, has improved the quality of life of the surgeon in such a way that it will eventually find its evidence in patient outcomes if studied further. On the other hand, the Achilles’ heel of the robotic platform lies in its learning curve and its dependence on procedural volume. This will subsequently enhance the overall feasibility of the surgical procedure. Especially, in the context of liver and pancreas resections, the accepted benchmark for assessing both short- and long-term outcomes are typically based on series including at least a few dozen patients. In time, the oncological advantages will follow as well, as seen in the REAL RCT study for robotic rectal cancer surgery.

## 7. Conclusions

The GI malignancy with the highest-quality evidence supporting the advantages of a robotic approach is colorectal surgery, specifically for rectal cancer. In colorectal surgeries, studies have demonstrated benefits in the quality of mesorectal specimens and improvement in urinary and sexual function outcomes. Furthermore, these advantages appear more distinct in specific subgroups, such as patients with obesity or male patients. The main robotic advantages found in all GI malignancy surgeries were: lower rate of conversion to open surgery, reduced hospital stay, and lower estimated blood loss. Some studies have reported a higher rate of negative margins and improved lymph node yield with the robotic approach. Nevertheless, the majority of available studies do not demonstrate improved long-term oncological outcomes in favor of the robotic approach. This is most likely attributable to the relatively short period of widespread robotic utilization, and such favorable results may emerge in future studies.

This review provides a comprehensive evaluation of the cost-effectiveness of robot-assisted versus conventional surgery, considering surgical fees, hospital stay duration, recovery time, and clinical outcomes. One of its objectives is to assist healthcare stakeholders in making informed decisions regarding the adoption of robotic surgery.

## Figures and Tables

**Table 1:** Summary of Key Randomized Controlled Trials (RCTs).

Trial Name	Year Published	Cancer Type	Comparison Groups	Key Findings
DIPLOMA [3]	2023	Pancreatic (Distal)	Minimally Invasive vs. Open	Non-inferiority in radical resection and lymph node yield. 3-day shorter ICU admission for MIS group.
LEOPARD [4]	2019	Pancreatic (Distal)	Minimally Invasive vs. Open	Reduced blood loss and time to functional recovery for MIS group. Achieved a similar radical resection rate.
EUROPA [5]	2024	Pancreatic (PD)	Robotic vs. Open	The robotic group had higher hospital costs. The robotic group had greater rates of clinically relevant pancreatic fistulas and delayed gastric emptying.
Lu et al. [8]	2021, 2024	Gastric (Distal)	Robotic vs. Laparoscopic	The robotic approach was associated with faster postoperative recovery, lower morbidity, and earlier initiation of adjuvant therapy. 3-year disease-free survival was significantly higher in the robotic group (85.8% vs. 73.2%).
Ojima et al. [9]	2021	Gastric	Robotic vs. Laparoscopic	Lower incidence of postoperative complications of Clavien–Dindo grade II or higher in the robotic group (8.8% vs. 19.7%).
ROC’N’ROLL [10]	2024	Liver	Robotic vs. Laparoscopic	No significant difference between the groups in perioperative outcomes or quality of life within 90 days.
ROLARR [13]	2017	Rectal	Robotic vs. Laparoscopic	No significant difference in the primary endpoint of conversion to open surgery.
REAL [14,15]	2022, 2025	Rectal (Mid/Low)	Robotic vs. Laparoscopic	The robotic group had less positive circumferential resection margins, fewer postoperative complications, and faster postoperative gastrointestinal recovery. The 3-year cumulative incidence of locoregional recurrence rate was significantly lower in patients undergoing robot-assisted surgery. Robotic patients also had significantly improved disease-free survival.

**Table 2:** Comparison of Key Oncological Outcomes.

Outcome Metric	Robotic Approach	Laparoscopic/Open Approach
Lymph Node Yield (Pancreatic)	13–20	9–15
Lymph Node Yield (Gastric)	34.5	26.6
Lymph Node Yield (Colorectal)	27	24
R0 Resection Rate (Pancreatic)	84.4–95%	80.1–83%

## Data Availability

No new data were created or analyzed in this study. Data sharing is not applicable to this article.
